# Art-induced psychological well-being: Individual traits shape the beneficial effects of aesthetic experiences

**DOI:** 10.1371/journal.pone.0332321

**Published:** 2025-11-14

**Authors:** Serena Castellotti, Elisa Gragnoli, Giada Baglioni, Roberta Criminisi, Barbara Giangrasso, Maria Michela Del Viva

**Affiliations:** 1 Department of Neurosciences, Psychology, Drug Research and Child Health (NEUROFARBA), University of Florence, Florence, Italy; 2 Department of Health Sciences (DSS), University of Florence, Florence, Italy; Vanderbilt University, UNITED STATES OF AMERICA

## Abstract

Since the beginning of the last century, a wide variety of studies have provided evidence of the role of art in improving health and well-being. In this study, we engaged 92 young adults in a guided tour of a contemporary art exhibition, centered around human freedom themes, offering an immersive multi-sensory experience. We aim to discover potential immediate beneficial effects of such aesthetic experience on anxiety, empathy, and compassion, and investigate how these effects relate to visitors’ psychological traits. We also studied whether individual characteristics could explain differences in visitors’ behavioral responses – i.e., visit time and post-visit evaluations. Prior to the experience, we collected participants’ information about their art preferences, interests, and expertise, and assessed their psychological traits of curiosity, openness to experience, anxiety, empathy, and compassion. The visit was led by an expert guide, and participants’ behavior was recorded through mobile eye tracking. Standardized self-report scales were administered before and after the visit to measure art-induced benefits on psychological well-being. An ad-hoc post-visit questionnaire, including several dimensions (beauty, understanding, satisfaction, etc.), was finally administered. Results showed that state anxiety decreased following the visit, particularly among visitors with a high trait of anxiety. Empathic and compassionate feelings increased after the visit, particularly among visitors with initially low empathic abilities and low compassion for humanity. Participants with higher curiosity and openness traits tended to spend more time engaging with the artworks and gave overall more positive evaluations. Higher art-related dimensions were linked to stronger emotional reactions and a greater sense of satisfaction and personal enrichment. Our findings provide additional evidence of the impact of art enjoyment on well-being. Art experiences centered around deeply emotional human themes may indeed reduce anxiety and enhance other-oriented feelings. Importantly, psychological traits define clusters of people who may benefit more from experiencing such art exhibitions.

## Introduction

Since the early 2000s, there has been a significant proliferation of neuroscientific studies within the field of neuroaesthetics. This complex discipline emerged with the primary aim of investigating the neural mechanisms underlying the appreciation of art. In this context, functional neuroimaging techniques have made it possible to identify which regions of the brain are most involved in the process of artistic perception [1–3]. In recent years, neuroaesthetics studies have expanded their scope, setting two main additional objectives: understanding how art can influence human cognition, emotions, and behavior [4–7], and investigating the beneficial effects of engagement with art [8–12]. In this regard, the calming effect of art enjoyment has been recognized by the World Health Organization [13] and recently reaffirmed by the Organization for Economic Co-operation and Development [14] as an important factor in improving the well-being of the population.

Psychological well-being refers to a state of thriving, flourishing, contentment, and overall good physical and mental health; it is the ability to effectively carry out daily tasks and maintain a positive self-perception, and is linked to positive emotions, life satisfaction, and altruistic behavior [15]. One of the core components of psychological well-being is the cultivation of positive relationships [16], which are underpinned by empathy and compassion. Empathy – the ability to share perspectives and understand the feelings of others – plays a crucial role in fostering positive social relationships [17,18]. Compassion, which can be enhanced by empathy, is typically defined as the motivation to help those in need [19] and care for others [20]. Thus, compassion encourages the provision of social support, which in turn strengthens interpersonal bonds [21].

Particularly relevant to the present study is, therefore, emerging evidence showing that aesthetic experiences can enhance positive feelings toward others [22] and reduce personal negative emotions [8–10,23], thereby contributing to overall psychological well-being. When discussing *aesthetic experience*, it is important to keep in mind that the term encompasses many different forms of art, for example, music [24] and dance [25]. Here, we focus specifically on visual art, narrowing our exploration to this domain only. In this regard, it has been found that observing works of art that address social or human themes, such as pain, loneliness, or joy, promotes empathy [26]. This effect has been explained by the so-called phenomenon of *emotional immersion*; that is, exhibitions that allow viewers to engage with the experiences and emotions of others, generate understanding of others [27], and finally foster empathetic feelings [26,28]. Also, aesthetic consumption of a work of art can serve as an “escape” from the daily context, offering individuals a mental break that reduces stress [10]. Museum visits specifically contribute to a decrease in perceived stress, thanks to their ability to stimulate a sense of calm and contemplation [9,29]. Emotions elicited by artworks can also support effective regulation of negative emotions [13]. Therefore, in this context, the concept of psychological well-being has been proposed as engagement with art not only improves mood but also fosters a deeper connection with oneself and others. These effects may be especially beneficial for individuals experiencing stress, anxiety, or reduced other-oriented feelings, as art provides a symbolic and soothing outlet that supports emotional regulation.

To understand the processes underlying the influence of art on human behavior, aesthetic experiences have been studied in laboratory settings [30–37], as well as in the real context in which they occur, namely the museum [38–49]. Many factors have been shown to influence the quality of an aesthetic experience, from the characteristics of the physical context [47,50] to the perception of authenticity of the artwork [51,52], to the personal characteristics of the viewer [53–59]. Among personal factors, the viewer’s expertise has been shown to be a key aspect that strongly influences aesthetic appreciation [60,61]. Research consistently shows that art experts significantly differ from novices not only in their art preferences [62] but also in their cognitive and emotional responses to art [63,64]. For instance, representational art elicited the strongest emotional responses in non-artists, whereas abstract and contemporary art was evaluated more emotionally by artists [65]. Experts tend to focus more on style and technical execution, whereas non-experts often base their judgments on the visible content and personal associations with the work [66]. As widely demonstrated, the experience of aesthetic pleasure depends on achieving a satisfactory cognitive understanding of the artwork, as is often the case for art experts. The greater the understanding, the lower the ambiguity, and the higher the likelihood of a positive aesthetic response [60,66].

Recent research has also increasingly focused on the relationship between art and personality, suggesting that psychological traits can shape artistic engagement [53,55,67]. For example, people with a high score of the Need for Cognitive Closure trait (NFC) spend less time viewing paintings before providing their liking ratings, reflecting their “tendency to urgency” [68]. Individuals with autistic traits show less distinction in their evaluations of high- and low-quality artworks, due to their weaker reliance on intuitive judgments and a stronger use of analytical thinking [69]. Also, it has been found that the trait of curiosity is a robust and reliable predictor of an individual’s eye movement behavior in scene-viewing; for example, people with a high trait of curiosity have been found to tend to view more areas of paintings [70]. Among the personality traits measured through the Big Five [71], openness to experience (OTE) has been recognized as a good predictor of people’s cultural behavior: people scoring high on openness are more likely to visit museums and to engage in other intellectual free-time activities [72–75]. Individuals with high OTE scores not only show a particular interest in visual arts and creative performances [76] but also experience more intense emotions when engaging with artworks [56,77,78]. Highly empathic people also reported enriched aesthetic experiences [79].

Building on the findings discussed so far, the overarching purpose of our research was to further investigate the potential beneficial effects of art enjoyment on psychological well-being and the relationship between these effects and visitors’ personal characteristics.

Although the concept of *well-being* in psychology and the social sciences is broad, encompassing various dimensions of human life such as physical, social, and economic aspects, in the context of this study, we specifically refer to the positive emotional and psychological sensations toward oneself and others that are induced by a cultural experience. In particular, we focus on a contemporary art exhibition centered on deep emotional themes to examine its effects on anxiety, compassion, and empathy.

### Description of the exhibition

The exhibition “*Libertà clandestine*” by Mariana Ferratto, an Italian Argentine artist, curated by Valentina Gensini, was held at the Murate Art District (MAD) in Florence. Housed in a former convent later converted into a city prison, MAD is now a cultural hub dedicated to contemporary artistic expressions (https://www.murateartdistrict.it/liberta-clandestine/). The exhibition in question engages with, narrates, and addresses the space of “clandestine creativity” established by Argentine political prisoners during the dictatorship from 1976 to 1983. The spaces of MAD thus perfectly accommodate and embrace the historical narratives presented in the exhibition. All artworks presented focused on how creativity and art fostered resilience and survival, helping prisoners endure the months and years spent in those cells. The exhibition showcased various forms of clandestine craftsmanship and communication methods used by prisoners. Visitors were guided through different areas, beginning with a display of handmade artifacts and a visual catalog illustrating symbols and gestures used for covert communication. Visitors then explored prison cells featuring tutorial videos demonstrating creative techniques developed by inmates using salvaged materials like bones, nails, and threads. Each station offered hands-on opportunities for visitors to replicate these crafts. The final part led participants to the high-security prison area, preserved in its original state for a more immersive experience. Here, they encountered artistic installations and audio recordings of prisoners sharing their experiences.

### The current study

The study involved 92 young participants in a guided tour of a contemporary art exhibition focused on the themes of human freedom. The visit provided an immersive multisensory experience incorporating several recognized health-promoting components, such as aesthetic engagement, imaginative involvement, cognitive stimulation, and emotional evocation [13]. We investigated how this experience, despite eliciting intense and sometimes negative emotional reactions, contributes to psychological well-being. To this end, we employed standardized self-report scales to assess changes in anxiety levels as well as in empathetic and compassionate feelings induced by the visit. We then explored how these effects relate to individual characteristics of the visitors. Finally, we studied how psychological traits, such as curiosity and openness to experience, and art-related factors, such as interest and expertise, influence visitors’ behavior within the exhibition, as recorded by portable eye-tracking glasses, and their final evaluation of the experience.

Our findings revealed a reduction in anxiety and an increase in other-oriented feelings following the visit. Moreover, psychological traits significantly influenced visit duration, behavioral responses, and, most importantly, the extent of the beneficial effects.

## Materials and methods

### Participants

Participants were recruited through social media posts published on the official profiles of our department and through paper-based flyers posted at the Faculty of Psychology of the University of Florence, inviting students to participate. The study was presented in general terms as an investigation in the field of art and neuroscience, explicitly informing participants that their involvement would require attending a contemporary art exhibition at the Murate Art District in Florence.

Ninety-two participants (25 men and 67 women), with an age range of 18–40 years (M = 24.1 years, SD = 4.0), took part in the study. All participants were naïve to the purpose of the study, and they had given written informed consent prior to participation. Experimental procedures were approved by the local ethics committee (“Commissione per l’Etica della Ricerca”, University of Florence, November 2, 2022, No. 229) and are in line with the Declaration of Helsinki. No one received monetary compensation for participation. All participants have normal or corrected-to-normal visual acuity, did not take any medications, did not present any brain damage, were free of cognitive disorders (e.g., ADHD, heart problems), and did not consume stimulant substances in the last 24 hours (e.g., caffeine, alcohol). Most of the participants were students (80% of our sample), 18% were workers, and 2% were unemployed. The education level was medium-high: 55% of our sample had a high-school diploma at the time of the experiment, 38% held a bachelor’s degree, 5% had a master’s degree, and 2% had a middle school diploma. The majority of our sample (74%) had a high school-level education in art history, only a few had a university-level education (8%), while the remaining participants had a middle school-level art history education (18%). Additionally, 23% of participants had attended art history courses, 17% had taken painting courses, and 37% engaged in artistic activities as a hobby, such as dance, painting, digital drawing, or ceramics.

Before data collection, participants were asked about their prior knowledge of the exhibition, the artist, and the museum. None of them had previously visited the exhibition or were familiar with the artist Mariana Ferratto. Additionally, only 18% of participants were aware of the historical events that took place during the Argentine dictatorship of 1976–1983, which was the theme of the exhibition. On average, participants’ expectations toward the exhibition were medium-high, with a mean score of 4.4 ± 1.1 on a 7-point Likert scale from 1 (“very low”) to 7 (“very high”). Furthermore, 13% of participants had visited the Murate Art District in Florence at least once, and 68% had attended a contemporary art exhibition or installation at least once in their lifetime.

Participants’ art-related characteristics were also evaluated by different questionnaires. Art Interest was measured with the Vienna Art Interest and Art Knowledge Questionnaire, specifically the Art Interest scale (Part A) (VAIAK; Specker et al., 2020). It included seven subjective interest items (e.g., “*I like to talk about art with friends and family*”) scored from 1 (“not at all”) to 7 (“very much”), and four art-interested behavior items (e.g., “*How often do you on average visit art museums*?), scored from 1 (“less than once a year”) to 7 (“once a week or more often”). The average interest score was 43.2 (SD = 1.3) over a maximum score of 77.

An ad-hoc questionnaire was used to assess participants’ art preferences and expertise. Participants were asked to rate some statements about their level of preference for different art styles (e.g., “*I like figurative art*”) on a 7-point Likert scale, from 1 (“not at all”) to 7 (“absolutely yes”). It turns out that our sample prefers figurative art (M = 5.14; SD = 0.2) to abstract art (M = 4.28; SD = 0.2). This is expected as non-expert people usually prefer figurative artworks compared to abstract ones [80–82]. Then, they were asked to self-evaluate their level of expertise in the field of art by indicating whether they considered themselves art experts or artists, using a 7-point Likert scale, ranging from 1 (“not at all”) to 7 (“absolutely yes”). The average scores were very low: most participants did not consider themselves art experts (M = 2.5, SD = 0.16) or artists (M = 2.2, SD = 0.15).

### Materials and set-up

#### Standardized self-report scales.

Curiosity was measured using the Curiosity and Exploration Inventory-II (CEI-II [83] – translated into Italian). It consists of 10 items assessing the motivation to seek out knowledge and new experiences (e.g., *“I actively seek as much information as I can in new situations”*) and the willingness to embrace the novel and unpredictable nature of everyday life (e.g., *“I view challenging situations as an opportunity to grow and learn”*). Respondents rate each item on a 5-point Likert scale from 1 (“very slightly or not at all”) to 5 (“extremely”), obtaining a total score ranging from 10 to 50.

Openness to experience (OTE) was measured using the Openness subscale from the Big Five Aspect Scales (BFAS [84] – translated into Italian). This subscale included 10 items testing the predisposition to be intellectually curious, open to emotion, sensitive to beauty, and willing to try new things (e.g., “*I see beauty in things that others might not notice*”). Respondents rate items using a 5-point Likert scale from 0 (“never” or “very rarely true”) to 4 (“very often” or “always true”), obtaining a total score ranging from 0 to 40.

Anxiety was measured using the State-Trait Anxiety Inventory (STAI [85]; Italian version by [86], which consists of two subscales: the STAI-T (Trait Anxiety) and the STAI-S (State Anxiety). The STAI-T subscale assesses trait anxiety, which reflects a person’s general tendency to experience anxiety. It includes 20 items (e.g., *“I worry too much over something that really doesn’t matter”*), to which respondents answer based on how they usually feel using a 4-point Likert scale from 1 (“almost never”) to 4 (“very much so”), obtaining a total score ranging from 20 to 80. The STAI-S subscale measures state anxiety, which refers to temporary feelings of anxiety experienced at a specific moment. It also consists of 20 items (e.g., *“I am tense”*, *“I feel calm”*), and respondents answer based on how they feel at that moment, using a 4-point Likert scale from 1 (“not at all”) to 4 (“very much”), obtaining a total score ranging from 20 to 80.

Empathy was measured using the Interpersonal Reactivity Index (IRI [87]; Italian version by [88]). It consists of 28 items, which are answered on a 5-point Likert scale ranging from 0 (“does not describe me well”) to 4 (“describes me well”). Items are divided into four subscales: “Perspective Taking” (PT), which is the tendency to spontaneously adopt the psychological point of view of others; “Fantasy” (FS), which is the ability to transpose oneself imaginatively into the feelings and actions of fictitious characters in books, movies, and plays; “Empathic Concern” (EC) which assesses “other-oriented” feelings of sympathy and concern for unfortunate others; and “Personal Distress” (PD) which measures “self-oriented” feelings of personal anxiety and unease in tense interpersonal settings. Each subscale is composed of 7 items, with a total score ranging from 0 to 28. A global empathy score is calculated by summing the scores of all subscales [89].

Compassion was measured with the Compassionate Love Scale for Humanity (CLS-H [90]; short Italian version by [91]). It consists of 21 items that evaluate the degree to which an individual feels compassion or altruistic love towards strangers, selfless caring, and the motivation to help humanity (e.g., “*It is easy for me to feel the pain and joy experienced by others, even though I do not know them*”). Participants rated each item on a 7-point Likert-type scale from 1 (“not at all true of me”) to 7 (“very true of me”), with a total score ranging from 10 to 80.

#### Ad-hoc post-visit questionnaire.

For gathering information about participants’ subjective evaluation of the experience at its end, we created an ad-hoc questionnaire comprising 24 statements covering several dimensions, such as appreciation (e.g., “I liked the visit”), understanding (“I understood the message of the exhibition”), emotions (“I felt positive emotions”), guide utility (“The educators’ explanations during the visit were helpful”), etc. All items were rated on a 7-points scale from 1 (“strongly disagree”) to 7 (“strongly agree”). The complete questionnaire can be found in S1 Table in S1 File.

#### Mobile eye-tracking.

Wearable eye-tracking glasses (Pupil Invisible, Pupil Labs, Berlin, Germany) enabled the recording of visitors’ behavior inside the exhibition, including time spent in front of different artworks, interactions with the guide, and visit paths. The eye tracker, consisting of two eye cameras (200 Hz) and a world camera (30 Hz), was connected to a dedicated Android device that participants carried in their pocket – the Pupil Invisible app was running, allowing real-time gaze estimation, recording, and streaming. Afterwards, recordings were uploaded to Pupil Cloud for data storage, visualization, and analysis.

### Procedure

The experimental procedure consisted of multiple phases. The first phase took place at home. Once participants were recruited, they received via email a series of documents to fill out online. They first signed an informed consent form and provided their personal details. Then, they completed questionnaires about their art-related information (see “*Participants*” section for details), along with a series of standardized self-report scales to assess their baseline psychological traits. These included the CEI-II, Openness – BFAS subscale, STAI-T, IRI, and CLS-H-SF (see “*Standardized self-report scales*” section for details).

On the designated day of participation, participants arrived at the Murate Art District (MAD). Upon arrival, they answered a set of questions regarding their prior knowledge of the exhibition, the artist, and the museum (see “*Participants*” section for details). These questions were not administered at home to prevent participants from searching for information online beforehand. They were then accompanied to a specific room where they were equipped with an eye-tracker headset and a wristband sensor and given time to familiarize themselves with the devices. The experimenter assisted in the calibration of the eye-tracker, which involved observing five specific points (five colored circles displayed on a white wall) while the experimenter monitored and adjusted the calibration as needed. Once calibration was completed, recording commenced. Finally, before beginning the guided tour, participants completed the STAI-S questionnaire to assess their anxiety state just prior to the visit (see “*Standardized self-report scales*” section for details).

**Fig 1:**
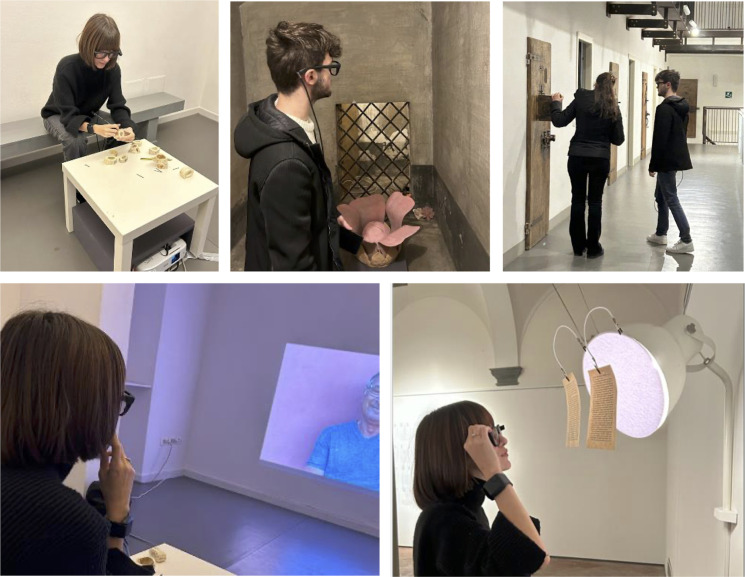
Pictures taken during the visit. From left to right, top to bottom: the participant learning how prisoners crafted small pendants from animal bones; the participant inside a prison cell installation, where the voice of a prisoner recounts their experience, transmitted through a terracotta flower on the floor; the participant engaged in a discussion with the guide in front a cell door; the participant watching a video projection; the participant interacting with an installation, decoding a poem by reading letters on hanging pages. Across all images, the eye tracker headset worn by the participants is visible. The photographs depict the exhibition *Libertà Clandestine* by Mariana Ferratto, curated by Valentina Gensini, held at MAD Murate Art District from October 19, 2023, to January 7, 2024 (details of the installations on display). The individuals pictured in Fig 1 have provided written informed consent (as outlined in the PLOS consent form) to publish their image alongside the manuscript.

After completing all pre-visit procedures, participants were left in the care of a museum educator and toured the exhibition individually under their guidance, while the experimenter remained in a dedicated room on the ground floor waiting for the tour to conclude. The educator led the participant to the first floor, where the exhibition began. As the study was conducted during regular opening hours, other visitors were occasionally present in the exhibition space. The visit started with a brief introduction to the exhibition’s theme and meaning, after which the educator guided participants through the artworks. The tour did not follow a fixed path but was instead adjusted based on each participant’s interests and preferences (Fig 1).

Once the guided tour ended, participants were free to continue exploring the exhibition independently. Many chose to revisit certain artworks or rewatch video projections, both on the first and second floors.

When participants decided to conclude their visit, they returned to the experimenter’s room on the ground floor; the recording was stopped, and the equipment was removed. Here, they re-administered the STAI-S, IRI, and CLS-H-SF tests to evaluate their post-visit anxiety, empathy, and compassion levels. Then, they filled out the post-visit questionnaire for the experience evaluation.

### Data processing and statistical analysis

#### Pre-visit vs. post-visit responses.

To assess changes in state anxiety, we analyzed the difference between pre-visit and post-visit scores on the STAI-S test. Data normality was verified using the Shapiro-Wilk test on the score differences. Since the assumption of normality was met (p > 0.05), we conducted a paired samples t-test to determine whether there was a significant difference between pre- and post-visit anxiety scores. The effect size was calculated using Cohen’s *d*.

Differences in participants’ scores on CLS-H-FS and IRI tests, completed before and after the visit, were analyzed to assess changes in compassion and empathy levels induced by the visit. Note that CLS-H-FS and IRI do not assess transient states but rather baseline levels of empathy and compassion. Therefore, the pre-visit scores, used to establish the participants’ reference levels, were collected at home before the experimental session. Since the pre-post difference scores for these measures did not follow a normal distribution (Shapiro-Wilk test, p < 0.05), we used the Wilcoxon signed-rank test. Effect sizes were calculated using rank-biserial correlation (r_rb_).

#### Correlations between individual characteristics and visit-induced effects.

For each participant, art-related information (see *Participants* section) was used to compute three main indices: an “art interest” score, corresponding to the VAIAK test (Part A; range: 11–77); an “aesthetic preference” score (range: 3–21), calculated as the sum of ratings for different art styles (figurative, abstract, contemporary); and an “art expertise” score (range: 2–14), derived from self-evaluations regarding being an art expert and an artist.

Differences between pre- and post-visit scores on the STAI-T, CLS-H-FS, and IRI – i.e., “visit-induced effects” – were correlated with all individual characteristics (art-related characteristics plus personal traits, i.e., CEI-II, OTE (BFAS subscale), STAI-T, CLS-H-FS, and IRI), using Spearman’s rank correlation for ordinal data.

#### Correlations between individual characteristics, visit time, and post-visit evaluations.

The time each participant spent inside the exhibition was obtained from the eye-tracker recordings by calculating the difference (in minutes) between the moment the educator started the introduction and the exact time the participant decided to conclude their visit.

For each participant, post-visit evaluations consisted of 24 scores (ranging from 1 to 7) across different dimensions of the final questionnaire (see S1 Table in S1 File).

Individual characteristics were correlated with visit duration and post-visit questionnaire item scores using Spearman’s rank correlation for ordinal data.

All statistical analyses were performed using JASP software (JASP team, Version 0.19.3, Release 2025, https://jasp-stats.org/).

## Results

### Pre-visit vs. post-visit responses

A key question of this study is whether the proposed aesthetic experience, despite addressing deep emotional themes, including those that may evoke negative emotions, ultimately led participants to “feel better”.

**Fig 2:**
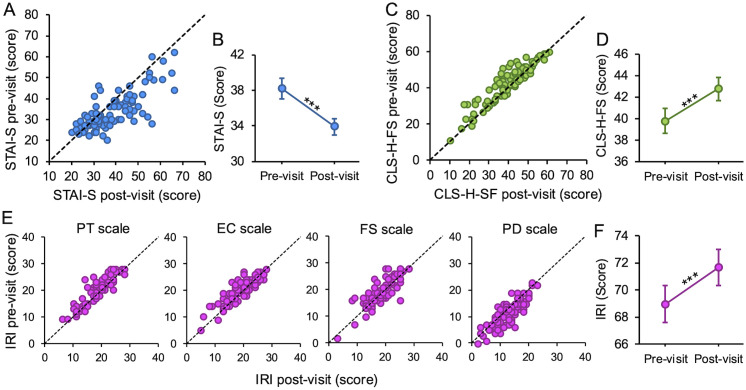
Visit-induced effects. **(A-B)** Anxiety state before and after the visit. **(B-C)** Compassion feelings before and after the visit. **(D-E)** Empathy feelings before and after the visit.

To investigate this, we first examined whether participants’ state anxiety significantly changed after the experience. Fig 2A displays individual STAI-T scores recorded immediately before and after the visit. Notably, for most participants, post-visit scores are lower than pre-visit scores. Fig 2B presents the mean scores across all participants (Pre-visit: M = 38.2, SE = 1.2; Post-visit: M = 33.9, SE = 0.9). Paired-samples t-test confirmed a significant decrease in state anxiety following the visit (t(91) = 5.68, *p* < 0.001, **d* *= 0.5 (0.07 SE) – *medium* effect).

Moreover, we wondered whether this particular exhibition might stimulate positive other-oriented feelings. To investigate this, we first examined whether participants’ compassion significantly changed after the experience. Fig 2C displays individual CLS-H-SF scores recorded before and after the visit. Notably, for most participants, post-visit scores are greater than pre-visit scores. Fig 2D presents the mean scores across all participants (Pre-visit: M = 39.8, SE = 1.1; Post-visit: M = 42.8, SE = 1.1). Wilcoxon signed-rank test confirmed a significant increase in compassionate feelings following the visit (W = 364.5, *p* < 0.001, r_rb_ = −0.75 (0.13 SE) – *large* effect).

Similar results emerged for empathetic feelings. Fig 2E displays individual IRI scores for all four IRI subscales recorded before and after the visit. For PT, EC, and FS scales, participants’ post-visit responses are higher, while for PD scales, they tend to decrease after the visit. Wilcoxon signed-rank tests confirmed a significant increase in perspective taking ability (PT scale – W = 374, *p* < 0.001, r_rb_ = −0.75 (0.13 SE) – *large* effect), empathic concern (EC scale – W = 664, *p* < 0.001, r_rb_ = 0.53 (0.13 SE) – *moderate* effect), and fantasy (FS scale – W = 927, *p* < 0.01, r_rb_ = −0.4 (0.12 SE) – *moderate* effect), while a significant decrease emerged for personal distress (PD scale – W = 2222.5, *p* < 0.001, r_rb_ = 0.44 (0.13 SE) – *moderate* effect). Fig 2F presents the mean global empathy scores across all participants (Pre-visit: M = 68.9, SE = 1.4; Post-visit: M = 71.7, SE = 1.3). Wilcoxon signed-rank test confirmed a significant increase in empathetic feelings following the visit (W = 893.5, **p* *< 0.001, r_rb_ = −0.49 (0.12 SE) – *moderate* effect).

### Individual characteristics and visit-induced effects

Based on the above results, we found beneficial effects in terms of reduced anxiety and enhanced other-oriented feelings. As the second main aim of this study, we investigated whether these benefits were consistent across participants or whether their magnitude was related to specific individual characteristics. To this end, we correlated the pre-post difference scores on the STAI-S, CLS-H-SF, and IRI measures (i.e., visit-induced effects) with participants’ art-related characteristics (interest, preferences, and expertise) and psychological traits (curiosity, openness to experience, trait anxiety, empathy, and compassion).

Table 1 reports descriptive statistics (means and standard deviations) for all individual characteristics and correlation results between them and changes in anxiety, empathy, and compassion levels induced by the visit.

**Table 1:** Visit-induced effects vs. individual characteristics (art-related information and personal traits).

		Anxiety state		Compassionate feelings		Empathetic feelings	
Individual characteristics	M ± SD	*rho*	*p*	*rho*	*p*	*rho*	*p*
**Art interest (VAIAK)**	43.2 ± 12.6	−0,039	0,712	0,183	0,081	0,048	0,653
**Aesthetic preference**	13.9 ± 3.9	−0,120	0,253	−0,046	0,662	0,042	0,688
**Art expertise**	4.6 ± 2.5	−0,075	0,479	0,231	0.027*	−0,040	0,702
**Curiosity (CEI-II)**	33.6 ± 6.7	0,070	0,507	0,089	0,401	−0,087	0,441
**Openness to Experience (BFAS)**	30.6 ± 6.1	−0,088	0,403	−0,017	0,870	0,039	0,716
**Anxiety (STAI-T)**	46.5 ± 10.7	−0,312	0.002**	0,059	0,573	−0,036	0,733
**Compassion (CLS-H-FS)**	39.8 ± 10.8	0,039	0,712	−0,326	0.002**	−0,097	0,360
**Empathy (IRI)**	72.9 ± 13.19	−0,141	0,180	−0,075	0,480	−0,342	<.001**

The first column reports means and standard deviations for each measure. The other columns report Spearman correlation results between individual characteristics and changes in anxiety, empathy, and compassion levels induced by the visit. Significant correlations are marked with asterisks: ***p < 0.001, **p < 0.01, *p < 0.05.

**Fig 3:**
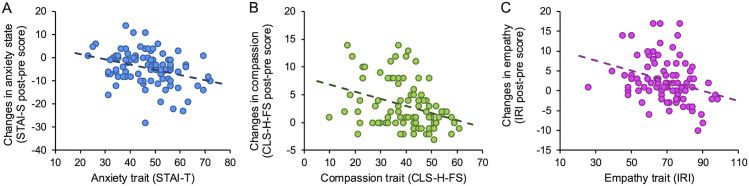
Psychological traits and visit-induced effects. **(A)** Changes in anxiety state vs. anxiety trait (rho = −0.312, p = 0.002). **(B)** Changes in compassionate feelings vs. compassion trait (rho = −0.326, p = 0.002) **(C)** Changes in empathetic feelings vs. empathy trait (rho = −0.342, p < 0.001).

Fig 3 displays significant correlations that emerged between psychological traits and visit-induced benefits. Benefits in terms of reduced state anxiety were associated with higher trait anxiety (Fig 3A), meaning that individuals with higher baseline anxiety experienced greater reductions. Greater benefits in compassionate feelings were instead negatively correlated with the compassion trait (Fig 3B); that is, individuals with lower dispositional compassion experienced stronger improvements in compassionate feelings after the visit. Similarly, greater benefits in empathic feelings were associated with lower empathy traits (Fig 3C). On the other hand, individuals with high baseline traits of compassion and empathy do not show any increase in other-oriented feelings and thus seem less affected by the visit experience. Visit-induced benefits are instead not correlated with curiosity and openness to experience traits.

As for art-related information, increases in compassion positively correlated with participants’ self-evaluation of expertise (graph not shown; see Table 1). Art interests and aesthetic preferences do not correlate with any benefits.

### Individual characteristics, visit time, and post-visit evaluations

As a final goal, we investigated whether participants’ individual characteristics could explain differences in their behavioral responses, specifically the time they spent inside the exhibition and their visit evaluations. The time participants dedicated to the visit varied considerably, ranging from fifteen minutes to over an hour (M = 35.5, SD = 12.3 min). All visitors gave overall positive judgments of the experience, reporting high levels of appreciation and satisfaction (see S1 Fig in S1 File for mean responses provided by participants for each item of the post-visit questionnaire).

Table 2 reports the correlation results between individual characteristics, visit duration, and all items of the post-visit questionnaire.

**Table 2:** Individual characteristics vs. visit time and post-visit evaluations.

		Art interest (VAIAK)		Aesthetic preference		Art expertise		Curiosity (CEI-II)		OTE (BFAS)		Anxiety (STAI-T)		Compassion (CLS-H-FS)		Empathy (IRI)	
		*rho*	*p*	*rho*	*p*	*rho*	*p*	*rho*	*p*	*rho*	*p*	*rho*	*p*	*rho*	*p*	*rho*	*p*
	**Visit time**	0.10	0.340	0.02	0.888	0.05	0.613	0.32	0.002**	0.51	< .001***	−0.04	0.725	0.05	0.661	−0.02	0.844
**Post-visit questionnaire items**	**1. Arousal**	0.23	0.025*	0.20	0.063	0.10	0.365	0.09	0.396	0.29	0.005**	−0.03	0.795	0.19	0.067	0.30	0.004**
	**2. Enjoyment**	0.44	< .001***	0.26	0.014*	0.37	< .001***	0.21	0.044*	0.30	0.004**	−0.16	0.120	0.03	0.794	0.06	0.593
	**3. Positive emotions**	0.27	0.011*	0.24	0.023*	0.14	0.171	0.27	0.01**	0.34	0.001**	−0.01	0.957	0.14	0.187	0.17	0.116
	**4. Negative emotions**	−0.07	0.482	−0.17	0.106	−0.14	0.198	0.02	0.825	0.12	0.255	0.18	0.090	−0.04	0.718	0.06	0.585
	**5. Beauty**	0.21	0.046*	0.08	0.46	0.17	0.098	0.04	0.725	0.14	0.181	−0.08	0.440	0.18	0.086	0.31	0.003**
	**6. Interest**	0.30	0.003**	0.18	0.094	0.18	0.088	0.17	0.106	0.32	0.002**	−0.09	0.420	0.27	0.01**	0.35	< .001***
	**7. Complexity**	0.07	0.518	0.07	0.53	−0.02	0.858	0.09	0.42	−0.04	0.693	−0.07	0.528	0.11	0.306	0.00	0.987
	**8. Understanding**	0.46	< .001***	0.25	0.016*	0.59	< .001***	0.21	0.047*	0.21	0.041*	−0.17	0.102	0.08	0.472	0.20	0.059
	**9. Curiosity**	0.38	< .001***	0.24	0.021*	0.22	0.033*	0.28	0.006**	0.35	< .001***	−0.06	0.583	0.19	0.078	0.23	0.026*
	**10. Expectation match**	0.17	0.098	0.16	0.12	0.02	0.861	0.03	0.768	0.17	0.106	−0.04	0.730	0.18	0.086	0.26	0.011*
	**11. Flow**	0.06	0.561	0.08	0.434	0.12	0.239	0.01	0.904	−0.02	0.838	−0.06	0.572	0.07	0.529	0.07	0.489
	**12. Multisensory stimulation**	0.12	0.243	0.05	0.643	0.07	0.503	0.08	0.428	0.29	0.005**	−0.12	0.246	0.04	0.686	0.18	0.088
	**13. Setting influence**	−0.05	0.632	−0.12	0.262	−0.09	0.402	0.07	0.489	−0.04	0.694	0.17	0.105	−0.07	0.524	−0.11	0.283
	**14. Title informativeness**	0.04	0.711	−0.15	0.154	0.06	0.58	0.09	0.416	0.21	0.047*	0.02	0.873	0.09	0.402	0.23	0.026*
	**15. Captions utility**	−0.12	0.27	−0.04	0.704	−0.12	0.27	−0.11	0.289	0.12	0.27	0.04	0.707	−0.11	0.283	0.01	0.923
	**16. Guide utility**	−0.01	0.897	0.07	0.51	−64.1	0.995	0.09	0.395	−0.01	0.91	−0.01	0.937	0.24	0.021*	0.23	0.029*
	**17. Environment influence**	0.20	0.061	0.07	0.526	0.08	0.426	0.08	0.44	0.06	0.556	−69.68	0.995	0.30	0.004**	0.27	0.008**
	**18. Virtual participation**	−0.02	0.845	−0.05	0.656	0.03	0.76	−0.15	0.142	−0.29	0.005**	0.17	0.105	−0.24	0.021*	−0.18	0.094
	**19. Empathy**	0.27	0.009**	0.25	0.017*	0.15	0.168	0.06	0.569	0.34	< .001***	0.04	0.727	0.36	< .001***	0.58	< .001***
	**20. Identification**	0.22	0.037*	0.187	0.074	0.13	0.231	0.10	0.367	0.39	< .001***	−0.02	0.878	0.30	0.003**	0.533	< .001***
	**21. Feeling lucky**	0.03	0.782	0.179	0.087	−0.05	0.629	0.07	0.518	0.10	0.352	−0.17	0.104	0.35	< .001***	0.358	< .001***
	**22. Enrichment**	0.26	0.012*	0.126	0.233	0.17	0.107	0.10	0.366	0.39	< .001***	−0.06	0.605	0.39	< .001***	0.430	< .001***
	**23. Feeling better**	0.20	0.051	0.156	0.136	0.14	0.198	0.16	0.132	0.18	0.08	0.06	0.565	0.09	0.389	0.179	0.089
	**24. Satisfaction**	0.33	0.001**	0.282	0.006**	0.27	0.011*	0.24	0.019*	0.39	< .001***	0.06	0.561	0.26	0.012*	0.346	< .001***

The third row shows Spearman correlation results between visit time and individual characteristics. The subsequent rows report Spearman correlations between individual characteristics and each item of the post-visit questionnaire (full item statements are listed in S1 Table in S1 File). Significant correlations are marked with asterisks: *p < .001, p < .01, p < .05.

**Fig 4:**
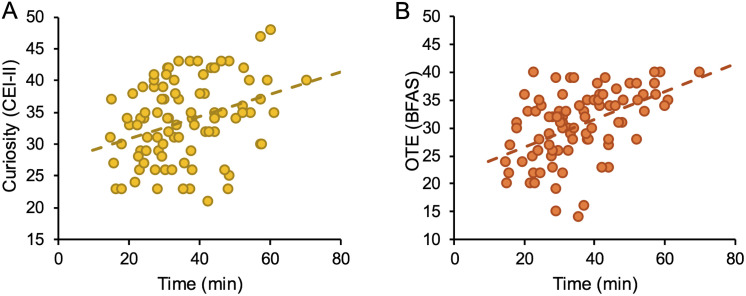
Psychological traits and visit time. **(A)** Curiosity trait and visit time (rho = −0.319, p = 0.002). **(B)** Openness-to-experience trait and visit time (rho = −0.509, p < 0.001).

Fig 4 displays the significant correlations that emerged between traits and visit duration; that is, participants with higher levels of curiosity and openness to experience traits tended to spend more time engaging with the artworks.

Individual characteristics also influence participants’ subjective evaluations (Table 2). Specifically, art-related characteristics – namely art interest, aesthetic preference, and art expertise – were significantly associated with more positive responses. Overall, higher scores in these dimensions were linked to stronger positive emotional reactions, greater enjoyment, and a better understanding of the exhibition’s message, leading to a deeper connection with the situations presented by the artist. These characteristics were also associated with higher levels of satisfaction and greater curiosity for future exhibitions.

Psychological traits also played a role (Table 2); participants with higher levels of curiosity and openness to experience reported significantly greater personal enrichment, satisfaction, and interest in future exhibitions, as well as deeper engagement with the content. These individuals also tended to evaluate the visit as more emotionally impactful and demonstrated a greater ability to focus on the artworks and the artist’s message without distractions. The anxiety trait was not associated with any dimension of the questionnaire. Finally, as expected, more empathic and compassionate individuals showed a stronger emotional involvement during the visit. This was reflected in more intense reactions, greater empathic resonance with the portrayed situations, and heightened self-reflective responses. These traits were also associated with increased satisfaction and a more profound sense of personal enrichment.

## Discussion

A wide variety of studies employing different methodologies indicated that engaging with art, in all its various forms, can improve both mental and physical health [8–12]. In this study, we further explore the positive effects of art experiences on psychological well-being by observing the behavior of 92 young participants during a contemporary art exhibition focused on the “clandestine creativity” of a group of individuals who were unjustly incarcerated. The exhibition took place in a museum that was formerly a prison, thereby creating a powerful and emotionally charged atmosphere.

A central focus of our study is the assessment of the impact of such an aesthetic experience on anxiety levels. Our findings show that visitors reported an immediate decrease in anxiety following the visit. This result aligns with previous works revealing the healing power of art [9,10,13,23,29]. However, this effect was not consistent across all participants: individuals with higher levels of trait anxiety experienced a more substantial reduction.

Given the nature of the exhibition selected for this study, we also investigated its potential to evoke positive affective responses toward others. First, we found an increase in compassion following the visit. This finding aligns with previous research demonstrating the pleasure of being moved in aesthetic experiences, even when we are exposed to artworks that portray sad content [92,93]. Compassion, understood as the ability to recognize the suffering of others along with the desire to alleviate it, can indeed evoke positive emotions even in contexts where it arises from depictions of pain or suffering. In these cases, viewing painful or tragic artworks not only leads to sadness but also fosters feelings of tenderness, hope, and a sense of shared humanity [94]. Furthermore, the broaden-and-build theory [95] suggests that positive emotions, such as those generated by compassion, broaden awareness and contribute to building long-term psychological resources. This mechanism may explain why art depicting suffering, when perceived through a compassionate lens, can contribute to the observer’s emotional growth and sense of well-being.

Also, we found an increase in empathy following the visit. Specifically, participants exhibited enhanced perspective-taking – reflecting a heightened ability to consider and adopt the viewpoints of others – as well as an increase in empathic concern, characterized by greater emotional sensitivity toward others’ experiences. We also observed a rise in fantasy, suggesting a greater tendency to imaginatively engage with hypothetical situations. Finally, a reduction in personal distress emerged, indicating lower levels of self-oriented discomfort compared to pre-visit assessments. Notably, the magnitude of these effects was related to participants’ baseline levels in each dimension: individuals with initially lower dispositional empathy and compassion benefited most from the experience. These findings further point to the potential value of similar cultural experiences as tools for emotional education, particularly for individuals with a low disposition toward understanding others’ feelings and limited motivation for social support [26–28].

On a more general ground, it is worth mentioning that our findings align with the foundational principles of art therapy, which draw on the intrinsic healing potential of artistic practice and use aesthetic engagement and creative expression to enhance emotional regulation [10,96,97]. In addition, our results resonate with key processes targeted by Acceptance and Commitment Therapy (ACT), cultivating the self as context and practicing cognitive defusion to reduce emotional avoidance and foster psychological flexibility [98,99]. The “imaginative immersion” elicited by art mirrors perspective-taking manipulations that can reduce personal distress and increase empathic concern [100]. Evidence from a museum-based social prescription intervention also suggests that guided visits can improve social connection and well-being in older adults, likely mediated by greater flexibility [101]. Taken together, these reflections highlight how even a brief museum visit can partially stimulate the same mechanisms promoted by ACT and emotional awareness interventions, fostering psychological flexibility, perspective-taking, and ultimately greater empathy and compassion.

Interestingly, the results indicate that observers with a higher level of expertise experienced greater benefits in terms of enhanced compassionate feelings. This effect may reflect experts’ superior ability to interpret the meaning of complex artistic styles, such as contemporary art [60], which may lead to deeper engagement with the artworks and a more nuanced understanding of the human conditions depicted. In turn, this deeper engagement could enhance the emotional resonance of the artworks and facilitate the emergence of compassionate feelings, highlighting the role of art expertise in maximizing the potential psychological benefits of museum experiences. The positive effects on anxiety and empathy do not appear to be linked to expertise, aesthetic preferences, or interest in art. However, this lack of association may also reflect the limited variability within our sample, as our participant pool consisted mostly of individuals with low levels of art expertise.

Our investigation employed a portable eye tracker to record visitor behavior inside the exhibition. Specifically, we measured visitor pathways, interactions with the guide, dwell time in different areas of the exhibition, and total visit duration. In this paper, we report data on the latter, as it showed considerable variability across participants and revealed meaningful correlations with their psychological traits. Participants were free to choose how much time to spend inside the museum: some dedicated only a few minutes to the experience, while others remained for over an hour, often engaging in hands-on activities or continuing the visit independently after the guided tour ended. Two psychological traits were found to influence this behavior: namely, curiosity and openness to experience. These traits have previously been identified as predictors of art appreciation [56,67,77].

Once participants decided to end their visit, they were asked to evaluate the exhibition across multiple dimensions (see S1 Table in S1 File for the full list of questions). Overall, they gave moderately high to very high ratings when evaluating their visit. The most appreciated aspect was the presence of an educator who accompanied them throughout the experience. This result confirms previous findings showing that guided interpretation played a central role in shaping participants’ engagement with the exhibition [102]. Indeed, the educator likely facilitated comprehension of complex content, stimulated curiosity through dialogue, and created a more personalized and interactive experience. This result highlights the importance of human mediation in cultural and educational settings: rather than being passive recipients of information, participants benefited from a socially embedded, interpretive experience. The second most appreciated element was the influence of the exhibition’s physical environment (see “Description of the exhibition” section for detailed descriptions about the museum setting). This result aligns with previous literature that highlights the importance of the physical context in which artworks are displayed [41,47–49,103]. On the other end, the lowest score was assigned to the item assessing whether a virtual version of the exhibition could be equally satisfying, again suggesting that participants strongly valued the physical and immersive nature of the in-person experience [31,32,41,47–49]. Participants also reported particularly positive impressions regarding the interest elicited by the exhibition, the personal enrichment derived from the experience, and their overall satisfaction. Notably, both positive and negative emotions were rated similarly high, further indicating that the positive outcomes described above were not simply the result of a pleasant experience. Rather, they appear to stem from a deep and emotionally engaging encounter, capable of evoking intense effects regardless of emotional valence.

Many studies have shown that art appreciation is strongly influenced by visitors’ art-related characteristics [62–65]. This trend also emerged in our data, as post-visit evaluations were particularly positive among individuals with a high interest in art, a strong aesthetic appreciation for diverse artistic styles, and a substantial level of art expertise. In particular, higher scores on the VAIAK questionnaire and higher self-assessments as experts or artists were significantly correlated with greater enjoyment of the visit, a deeper understanding of the exhibition’s message, and increased curiosity for future exhibitions. These findings thus confirm that pre-existing dispositions toward art can meaningfully shape the visitor’s emotional and cognitive engagement, ultimately enriching the overall museum experience and fostering a lasting interest in the artist’s work [65].

Psychological traits also play a significant role in shaping visit evaluations. As expected, individuals with higher compassion and empathy traits report greater cognitive and emotional connection with the exhibition content [26,79]. Individuals with high levels of curiosity reported a greater interest in attending future exhibitions, reflecting their natural tendency toward exploration and receptiveness to novelty [73,75]. The personality trait of openness to experience was positively correlated with nearly all evaluation scores [72]. This finding is consistent with previous research showing that OTE is a strong predictor of art appreciation [55,77]; individuals high in openness are indeed more likely to seek out and appreciate complex and emotionally rich stimuli, such as those found in artistic contexts [73,74,76]. In this sense, openness does not merely reflect a passive preference for art but rather an active engagement that deepens the overall aesthetic experience [56,77].

### Limitations of the study and future avenues

Some limitations of the current study stem from the specificity of the exhibition we selected, which featured characteristics that may not generalize to all museum contexts, thus leading to outcomes that may not automatically extend to more classical settings.

First, we cannot determine whether the positive outcomes we observed would have emerged in the absence of a dedicated museum educator. As previously discussed, understanding artworks is essential to appreciation, and guided facilitation has likely played a crucial role in enhancing participants’ satisfaction, as confirmed by their final evaluations. Moreover, the educator did not follow a fixed path but instead adapted the visit to the participant’s reactions and interests. Future studies could specifically examine whether this personalized format was crucial to the observed benefits, or whether similar outcomes could be achieved through a standardized and predetermined tour. It would also be valuable to design dedicated experiments to investigate whether comparable effects emerge during unguided visits, or visits accompanied by peers rather than expert guides, as well as to compare outcomes between educator-led visits and those supported by less interactive tools, such as audio guides.

Another distinctive element of the exhibition was its multisensory and interactive nature, which included hands-on activities rarely found in conventional museum visits. The immersive quality of the experience may have amplified its positive effects, which might not emerge in more passive, artwork-by-artwork visits.

The specific theme of the exhibition may also have contributed to the psychological benefits documented, particularly those related to empathy and compassion. Future research should assess whether similar effects arise in exhibitions centered on different, less emotionally evocative, topics. On a more general ground, classic museums usually exhibit several artworks that do not follow a unifying narrative; thus, replicating the current measures in more traditional settings could help clarify which components of the experience drive psychological well-being. While it is possible that such settings may not influence interpersonal feelings to the same extent, we may still observe reductions in anxiety following the visit.

A further potential development of this research would be to replicate the experiment with a sample of expert participants, such as artists, curators, or art historians. This would allow for a deeper understanding of the relative contribution of personal traits and acquired knowledge in shaping psychophysiological responses to aesthetic experiences.

One more limitation of the present study lies in the uncertainty regarding the duration of the beneficial effects observed. It remains unclear whether these benefits persist over time or tend to fade shortly after the visit. A reliable assessment of long-term outcomes would require a longitudinal approach, which was not feasible in the current work and would be subjected to numerous intervening variables that could complicate interpretation. Future dedicated studies should explore whether a single museum visit can induce sustained psychological benefits or whether repeated visits are needed to maintain these effects; a kind of dose–response pattern that may help clarify how frequently people should engage with art experiences to foster their well-being.

Finally, future works should further expand the investigation of art-induced benefits by exploring whether aesthetic engagement can also influence cognitive domains, such as cognitive flexibility, inhibitory control, and attentional processes.

## Conclusions

The present findings highlight the meaningful psychological benefits that can arise from engaging with contemporary art exhibitions. In particular, exposure to artworks centered on deeply emotional and human themes was associated with a reduction in anxiety and an increase in empathetic and compassionate feelings toward others. It is worth noting that, in principle, the exhibition in question may have elicited opposite reactions in some individuals, due to the intense themes it addressed, including isolation, physical and psychological suffering, and the struggle for survival. Instead, our data show that being emotionally moved by art experiences evoking strong sensations, regardless of whether they are positive or negative, still produces beneficial effects.

Both art-related expertise and individual psychological traits played a crucial role in shaping participants’ behavioral and emotional responses during the visit. Notably, psychological traits also influenced the extent to which individuals derived value from the experience.

In conclusion, art can be considered a powerful tool for fostering positive emotional responses toward oneself and others, ultimately contributing to psychological well-being. Understanding the role of individual differences may help identify clusters of people who are more likely to benefit from such aesthetic experiences. To fully harness these benefits, museum institutions could consider developing personalized pathways and targeted visits tailored to visitors’ characteristics, thereby maximizing the emotional and psychological impact of art engagement.

## Supporting information

S1 File S1 Table. Ad-hoc questionnaire for evaluation of the visit. The table shows the questionnaire items, in their original Italian version (first column) and their English translation (second column), in the order they were presented to the participants. Participants had to evaluate each statement on a Likert scale from 1 to 7, where 1 corresponded to “Strongly disagree,” 2 to “Disagree,” 3 to “Somewhat disagree,” 4 to “Neither agree nor disagree,” 5 to “Somewhat agree,” 6 to “Agree,” and 7 to “Strongly agree”. S1 Fig. Post-visit evaluations. Each bar shows the mean score for an item from the post-visit questionnaire (full item statements are listed in S1 Table in S1 File). Items are arranged in descending order based on their scores.
(DOCX)

## References

[1] Cela-Conde CJ, Marty G, Maestú F, Ortiz T, Munar E, Fernández A, et al. Activation of the prefrontal cortex in the human visual aesthetic perception. Proc Natl Acad Sci U S A. 2004;101(16):6321–5. doi: 10.1073/pnas.0401427101 15079079 PMC395967

[2] Ishizu T, Zeki S. Toward a brain-based theory of beauty. PLoS One. 2011;6(7):e21852. doi: 10.1371/journal.pone.0021852 21755004 PMC3130765

[3] Cattaneo Z, Ferrari C, Schiavi S, Alekseichuk I, Antal A, Nadal M. Medial prefrontal cortex involvement in aesthetic appreciation of paintings: a tDCS study. Cogn Process. 2020;21(1):65–76. doi: 10.1007/s10339-019-00936-9 31637555

[4] Gallese V. Embodied Simulation. Its Bearing on Aesthetic Experience and the Dialogue Between Neuroscience and the Humanities. Gestalt Theory. 2019;41(2):113–27. doi: 10.2478/gth-2019-0013

[5] Magsamen S. Your aesthetic brain: A growing case for the arts. Cerebrum.

[6] Pearce MT, Zaidel DW, Vartanian O, Skov M, Leder H, Chatterjee A, et al. Neuroaesthetics: The Cognitive Neuroscience of Aesthetic Experience. Perspect Psychol Sci. 2016;11(2):265–79. doi: 10.1177/1745691615621274 26993278

[7] Marković S. Components of aesthetic experience: aesthetic fascination, aesthetic appraisal, and aesthetic emotion. Iperception. 2012;3(1):1–17. doi: 10.1068/i0450aap 23145263 PMC3485814

[8] Zhang Y. Impact of arts activities on psychological well-being: Emotional intelligence as mediator and perceived stress as moderator. Acta Psychol (Amst). 2025;254:104865. doi: 10.1016/j.actpsy.2025.104865 40049081

[9] Thomson L, Chatterjee H. Assessing well-being outcomes for arts and heritage activities: Development of a Museum Well-being Measures toolkit. J Appl Arts Health. 2014;5(1):29–50. doi: 10.1386/jaah.5.1.29_1

[10] Law M, Karulkar N, Broadbent E. Evidence for the effects of viewing visual artworks on stress outcomes: a scoping review. BMJ Open. 2021;11(6):e043549. doi: 10.1136/bmjopen-2020-043549 34193477 PMC8246362

[11] Chatterjee H, Noble G. Museums, health and well-being. 2016. Epub ahead of print 2016. doi: 10.4324/9781315596549

[12] Fares J, Hadjicosti I, Constantinou C. Rethinking culture: a narrative review on the evolving role of museum and art gallery-based heritage activities and programmes on wellbeing. Perspect Public Health. 2024;1–13. 10.1177/17579139241268446 PMC12231841 39329522

[13] Fancourt D, Finn S. Health evidence network synthesis report 67: What is the evidence on the role of the arts in improving health and well-being? A scoping review. Copenhagen: WHO Regional Office for Europe; 2019. 32091683

[14] Travkina E, Sacco PL. Culture shock: COVID-19 and the cultural and creative sectors. OECD; 2020.

[15] Mota E, Brandão T, Costa SR. Understanding happiness among university students: The role of general health, psychological well-being, and sociodemographic variables. Mediterr J Clin Psychol. 2023;11. doi: 10.13129/2282-1619/mjcp-3589

[16] Ryff CD. Psychological well-being revisited: advances in the science and practice of eudaimonia. Psychother Psychosom. 2014;83(1):10–28. doi: 10.1159/000353263 24281296 PMC4241300

[17] Kardos P, Leidner B, Pléh C, Soltész P, Unoka Z. Empathic people have more friends: Empathic abilities predict social network size and position in social network predicts empathic efforts. Soc Networks. 2017;50:1–5. doi: 10.1016/j.socnet.2017.01.004

[18] Sened H, Lavidor M, Lazarus G. Empathic accuracy and relationship satisfaction: A meta-analytic review. J Fam Psychol. 2023. 10.1037/fam0000320 28394141

[19] Goetz JL, Keltner D, Simon-Thomas E. Compassion: an evolutionary analysis and empirical review. Psychol Bull. 2010;136(3):351–74. doi: 10.1037/a0018807 20438142 PMC2864937

[20] Gilbert P. Explorations into the nature and function of compassion. Curr Opin Psychol. 2019;28:108–14. doi: 10.1016/j.copsyc.2018.12.002 30639833

[21] Karnaze MM, Bloss CS. Protective roles of empathy and compassion against loneliness early in the SARS-CoV-2 pandemic. Curr Res Behav Sci. 2023;5:100130.

[22] Cotter KN, Mahlobo CT, Smith B, Niepold S, Rizzo A, Pawelski JO. Examining the Ability of Digital Visual Art Engagement to Cultivate Empathy and Social Connection. Empir Stud Arts. 2025;43(2):921–46. doi: 10.1177/02762374241309878

[23] Creech A, Hallam S, Varvarigou M, McQueen H, Gaunt H. Active music making: a route to enhanced subjective well-being among older people. Perspect Public Health. 2013;133(1):36–43. doi: 10.1177/1757913912466950 23308006

[24] Brattico E, Bogert B, Jacobsen T. Toward a neural chronometry for the aesthetic experience of music. Front Psychol. 2013;4:206. doi: 10.3389/fpsyg.2013.00206 23641223 PMC3640187

[25] Laird KT, Vergeer I, Hennelly SE, Siddarth P. Conscious dance: Perceived benefits and psychological well-being of participants. Complement Ther Clin Pract. 2021;44:101440. doi: 10.1016/j.ctcp.2021.101440 34260998

[26] Pelowski M, Markey PS, Forster M, Gerger G, Leder H. Move me, astonish me… delight my eyes and brain: The Vienna Integrated Model of top-down and bottom-up processes in Art Perception (VIMAP) and corresponding affective, evaluative, and neurophysiological correlates. Phys Life Rev. 2017;21:80–125. doi: 10.1016/j.plrev.2017.02.003 28347673

[27] Freedberg D, Gallese V. Motion, emotion and empathy in esthetic experience. Trends Cogn Sci. 2007;11(5):197–203. doi: 10.1016/j.tics.2007.02.003 17347026

[28] Wright AC. Art therapy with an autistic person with learning disabilities: communication and emotional regulation. Int J Art Ther. 2023;28(4):154–66. doi: 10.1080/17454832.2023.2172439

[29] Mastandrea S, Maricchiolo F, Carrus G, Giovannelli I, Giuliani V, Berardi D. Visits to figurative art museums may lower blood pressure and stress. Arts Health. 2019;11(2):123–32. doi: 10.1080/17533015.2018.1443953 31038442

[30] Mastandrea S, Bartoli G, Carrus G. The automatic aesthetic evaluation of different art and architectural styles. Psychol Aesthetics Creat Arts. 2011;5(2):126–34. doi: 10.1037/a0021126

[31] Bertamini M, Blakemore C. Seeing a Work of Art Indirectly: When a Reproduction Is Better Than an Indirect View, and a Mirror Better Than a Live Monitor. Front Psychol. 2019;10. doi: 10.3389/fpsyg.2019.02033 PMC6746833 31551877

[32] Castellotti S, D’Agostino O, Del Viva MM. Effectiveness of labels in digital art experience: psychophysiological and behavioral evidence. Front Psychol. 2024;15:1342667. doi: 10.3389/fpsyg.2024.1342667 39011289 PMC11248719

[33] Castellotti S, Conti M, Feitosa-Santana C, Del Viva MM. Pupillary response to representations of light in paintings. J Vis. 2020;20(10):14. doi: 10.1167/jov.20.10.14 33052409 PMC7571318

[34] Castellotti S, Scipioni L, Mastandrea S, Del Viva MM. Pupil responses to implied motion in figurative and abstract paintings. PLoS One. 2021;16(10):e0258490. doi: 10.1371/journal.pone.0258490 34634092 PMC8504727

[35] Nadal M, Munar E, Marty G, Cela-Conde CJ. Visual Complexity and Beauty Appreciation: Explaining the Divergence of Results. Empir Stud Arts. 2010;28(2):173–91. doi: 10.2190/em.28.2.d

[36] Massaro D, Savazzi F, Di Dio C, Freedberg D, Gallese V, Gilli G, et al. When Art Moves the Eyes: A Behavioral and Eye-Tracking Study. PLoS ONE. 2012;7(5):e37285. doi: 10.1371/journal.pone.0037285 PMC3356266 22624007

[37] Leder H, Carbon C-C, Ripsas A-L. Entitling art: Influence of title information on understanding and appreciation of paintings. Acta Psychol (Amst). 2006;121(2):176–98. doi: 10.1016/j.actpsy.2005.08.005 16289075

[38] Mastandrea S, Maricchiolo F, Carrus G, Giovannelli I, Giuliani V, Berardi D. Visits to figurative art museums may lower blood pressure and stress. Arts Health. 2019;11(2):123–32. doi: 10.1080/17533015.2018.1443953 31038442

[39] Castellotti S, D’Agostino O, Mencarini A, Fabozzi M, Varano R, Mastandrea S, et al. Psychophysiological and behavioral responses to descriptive labels in modern art museums. PLoS One. 2023;18(5):e0284149. doi: 10.1371/journal.pone.0284149 37134073 PMC10155981

[40] Carbon C-C. Art Perception in the Museum: How We Spend Time and Space in Art Exhibitions. Iperception. 2017;8(1):2041669517694184. doi: 10.1177/2041669517694184 28321289 PMC5347319

[41] Specker E, Tinio PPL, van Elk M. Do you see what I see? An investigation of the aesthetic experience in the laboratory and museum. Psychol Aesthetics Creat Arts. 2017;11(3):265–75. doi: 10.1037/aca0000107

[42] Reitstätter L, Galter K, Bakondi F. Looking to Read: How Visitors Use Exhibit Labels in the Art Museum. Visitor Studies. 2022;25(2):127–50. doi: 10.1080/10645578.2021.2018251

[43] Pelowski M, Forster M, Tinio PPL, Scholl M, Leder H. Beyond the lab: An examination of key factors influencing interaction with ‘real’ and museum-based art. Psychol Aesthetics Creat Arts. 2017;11(3):245–64. doi: 10.1037/aca0000141

[44] Reitstätter L, Brinkmann H, Santini T, Specker E, Dare Z, Bakondi F, et al. The Display Makes a Difference: A Mobile Eye Tracking Study on the Perception of Art Before and After a Museum’s Rearrangement. J Eye Mov Res. 2020;13(2):10.16910/jemr.13.2.6. doi: 10.16910/jemr.13.2.6 33828792 PMC7962802

[45] Rainoldi M, Neuhofer B, Jooss M. Mobile Eyetracking of Museum Learning Experiences. 2018. Epub ahead of print 1 January 2018. doi: 10.1007/978-3-319-72923-7_36

[46] Rainoldi M, Yu CE, Neuhofer B. The museum learning experience through the visitors’ eyes: An eye tracking exploration of the physical context. 2020. p. 183–99.

[47] Brieber D, Nadal M, Leder H, Rosenberg R. Art in time and space: context modulates the relation between art experience and viewing time. PLoS One. 2014;9(6):e99019. doi: 10.1371/journal.pone.0099019 24892829 PMC4043844

[48] Grüner S, Specker E, Leder H. Effects of Context and Genuineness in the Experience of Art. Empir Stud Arts. 2019;37(2):138–52. doi: 10.1177/0276237418822896

[49] Brieber D, Nadal M, Leder H. In the white cube: museum context enhances the valuation and memory of art. Acta Psychol (Amst). 2015;154:36–42. doi: 10.1016/j.actpsy.2014.11.004 25481660

[50] Szubielska M, Imbir K, Szymańska A. The influence of the physical context and knowledge of artworks on the aesthetic experience of interactive installations. Curr Psychol. 2019;40(8):3702–15. doi: 10.1007/s12144-019-00322-w

[51] Locher P, Smith L, Smith J. Original Paintings versus Slide and Computer Reproductions: A Comparison of Viewer Responses. Empir Stud Arts. 1999;17(2):121–9. doi: 10.2190/r1wn-taf2-376d-efuh

[52] Grassini S, Koivisto M. Understanding how personality traits, experiences, and attitudes shape negative bias toward AI-generated artworks. Sci Rep. 2024;14(1):4113. doi: 10.1038/s41598-024-54294-4 38374175 PMC10876601

[53] Cleridou K, Furnham A. Personality Correlates of Aesthetic Preferences for Art, Architecture, and Music. Empir Stud Arts. 2014;32(2):231–55. doi: 10.2190/em.32.2.f

[54] Packer J, Ballantyne R. Conceptualizing the visitor experience: a review of literature and development of a multifaceted model. Visit Stud. 2016;19:128–43.

[55] Afhami R, Mohammadi-Zarghan S. The Big Five, Aesthetic Judgment Styles, and Art Interest. Eur J Psychol. 2018;14(4):764–75. doi: 10.5964/ejop.v14i4.1479 30555584 PMC6266521

[56] Palumbo L, Harrison NR, Trawiński T, Kass J, Metelmann AC, Bari RSG, et al. Visual exploration mediates the influence of personal traits on responses to artworks in an art gallery setting. Psychol Aesthetics Creat Arts. 2025;19(2):270–83. doi: 10.1037/aca0000529

[57] Kenett YN, Humphries S, Chatterjee A. A thirst for knowledge: grounding curiosity, creativity, and aesthetics in memory and reward neural systems. Creat Res J. 2023;35:412–26.

[58] Trawiński T, Palumbo L, Begum R, Donnelly N. The effect of social factors on eye movements made when judging the aesthetic merit of figurative paintings. Sci Rep. 2024;14(1):21843. doi: 10.1038/s41598-024-72810-4 39294260 PMC11410938

[59] Pihko E, Virtanen A, Saarinen V-M, Pannasch S, Hirvenkari L, Tossavainen T, et al. Experiencing art: the influence of expertise and painting abstraction level. Front Hum Neurosci. 2011;5:94. doi: 10.3389/fnhum.2011.00094 21941475 PMC3170917

[60] Leder H, Belke B, Oeberst A, Augustin D. A model of aesthetic appreciation and aesthetic judgments. Br J Psychol. 2004;95(Pt 4):489–508. doi: 10.1348/0007126042369811 15527534

[61] Bianchi I, Burro R, Verstegen I, Branchini E, Bertamini M. Cognitive and historical information can spark interest in modern and contemporary art. Psychol Aesthetics Creat Arts. 2025. doi: 10.1037/aca0000764

[62] van Paasschen J, Bacci F, Melcher DP. The Influence of Art Expertise and Training on Emotion and Preference Ratings for Representational and Abstract Artworks. PLoS One. 2015;10(8):e0134241. doi: 10.1371/journal.pone.0134241 26244368 PMC4526639

[63] Bimler DL, Snellock M, Paramei GV. Art expertise in construing meaning of representational and abstract artworks. Acta Psychol (Amst). 2019;192:11–22. doi: 10.1016/j.actpsy.2018.10.012 30390421

[64] Darda KM, Cross ES. The role of expertise and culture in visual art appreciation. Sci Rep. 2022;12(1):10666. doi: 10.1038/s41598-022-14128-7 35739137 PMC9219380

[65] Else JE, Ellis J, Orme E. Art expertise modulates the emotional response to modern art, especially abstract: an ERP investigation. Front Hum Neurosci. 2015;9:525. doi: 10.3389/fnhum.2015.00525 27242497 PMC4876367

[66] Leder H, Gerger G, Dressler SG, Schabmann A. How art is appreciated. Psychol Aesthetics Creat Arts. 2012;6(1):2–10. doi: 10.1037/a0026396

[67] Chamorro-Premuzic T, Burke C, Hsu A, Swami V. Personality predictors of artistic preferences as a function of the emotional valence and perceived complexity of paintings. Psychol Aesthetics Creat Arts. 2010;4(4):196–204. doi: 10.1037/a0019211

[68] Ostrofsky J, Shobe E. The Relationship Between Need for Cognitive Closure and the Appreciation, Understanding, and Viewing Times of Realistic and Nonrealistic Figurative Paintings. Empir Stud Arts. 2015;33(1):106–13. doi: 10.1177/0276237415570016

[69] Brosnan M, Ashwin C. Differences in Art Appreciation in Autism: A Measure of Reduced Intuitive Processing. J Autism Dev Disord. 2023;53(11):4382–9. doi: 10.1007/s10803-022-05733-6 36063312 PMC10539443

[70] Risko EF, Anderson NC, Lanthier S, Kingstone A. Curious eyes: individual differences in personality predict eye movement behavior in scene-viewing. Cognition. 2012;122(1):86–90. doi: 10.1016/j.cognition.2011.08.014 21983424

[71] McCrae RR, Costa PT. The Five Factor Theory of Personality. Handbook of Personality. 2008.

[72] McCrae RR. Aesthetic chills as a universal marker of openness to experience. Motiv Emot. 2007;31(1):5–11.

[73] Meyer J, Thoma G-B, Kampschulte L, Köller O. Openness to experience and museum visits: Intellectual curiosity, aesthetic sensitivity, and creative imagination predict the frequency of visits to different types of museums. J Res Pers. 2023;103:104352. doi: 10.1016/j.jrp.2023.104352

[74] Schwaba T, Luhmann M, Denissen JJA, Chung JM, Bleidorn W. Openness to experience and culture-openness transactions across the lifespan. J Pers Soc Psychol. 2018;115(1):118–36. doi: 10.1037/pspp0000150 28557472

[75] Fayn K, MacCann C, Tiliopoulos N, Silvia PJ. Aesthetic Emotions and Aesthetic People: Openness Predicts Sensitivity to Novelty in the Experiences of Interest and Pleasure. Front Psychol. 2015;6:1877. doi: 10.3389/fpsyg.2015.01877 26696940 PMC4673303

[76] Silvia PJ, Nusbaum EC, Berg C. Openness to experience, plasticity, and creativity: exploring lower-order, high-order, and interactive effects. J Res Pers. 2009;43:1087–90.

[77] Chamorro-Premuzic T, Reimers S, Hsu A, Ahmetoglu G. Who art thou? Personality predictors of artistic preferences in a large UK sample: the importance of openness. Br J Psychol. 2009;100(Pt 3):501–16. doi: 10.1348/000712608X366867 19026107

[78] Rodriguez RM, Fekete A, Silvia PJ, Cotter KN. The art of feeling different: Exploring the diversity of emotions experienced during an art museum visit. Psychol Aesthetics Creat Arts. 2024;18(3):303–14. doi: 10.1037/aca0000443

[79] Wilkinson Z, Cunningham R, Elliott MA. The influence of empathy on the perceptual response to visual art. Psychol Aesthetics Creat Arts. 2024;18(3):259–68. doi: 10.1037/aca0000418

[80] Mastandrea S, Bartoli G, Bove G. Learning through Ancient Art and Experiencing Emotions with Contemporary Art: Comparing Visits in Two Different Museums. Empir Stud Arts. 2007;25(2):173–91. doi: 10.2190/r784-4504-37m3-2370

[81] Mastandrea S, Bartoli G, Bove G. Preferences for ancient and modern art museums: Visitor experiences and personality characteristics. Psychol Aesthetics Creat Arts. 2009;3(3):164–73. doi: 10.1037/a0013142

[82] Szubielska M, Francuz P, Niestorowicz E. The impact of reading or listening to a contextual information relating to contemporary paintings on the evaluation by non-experts in the field of art. Polish Psychol Forum. 2018;23:610–27.

[83] Kashdan TB, Gallagher MW, Silvia PJ, Winterstein BP, Breen WE, Terhar D, et al. The Curiosity and Exploration Inventory-II: Development, Factor Structure, and Psychometrics. J Res Pers. 2009;43(6):987–98. doi: 10.1016/j.jrp.2009.04.011 20160913 PMC2770180

[84] DeYoung CG, Quilty LC, Peterson JB. Between facets and domains: 10 aspects of the Big Five. J Pers Soc Psychol. 2007;93(5):880–96. doi: 10.1037/0022-3514.93.5.880 17983306

[85] Spielberger CD. State-Trait Anxiety Inventory: Bibliography (2nd edition). 1989.

[86] Pedrabrissi Santinello ML. Verifica della validità dello STAI forma Y di Spielberger. Giunti Organ Spec. 2023;15.

[87] Davis MH. A multidimensional approach to individual differences in empathy. J Pers Soc Psychol. 1983;44:113–26.

[88] Albiero P, Ingoglia S, Lo Coco A. Contributo all’adattamento italiano dell’interpersonal reactivity index di Davis. TPM. 2006;13(2):107–25.

[89] Eerola T, Vuoskoski JK, Kautiainen H. Being Moved by Unfamiliar Sad Music Is Associated with High Empathy. Front Psychol. 2016;7:1176. doi: 10.3389/fpsyg.2016.01176 27695424 PMC5025521

[90] Sprecher S, Fehr B. Compassionate love for close others and humanity. J Soc Pers Relat. 2005;22:629–51.

[91] Chiesi F, Lau C, Saklofske DH. A revised short version of the compassionate love scale for humanity (CLS-H-SF): evidence from item response theory analyses and validity testing. BMC Psychol. 2020;8(1):20. doi: 10.1186/s40359-020-0386-9 32087755 PMC7036195

[92] Hanich J, Wagner V, Shah M, Jacobsen T, Menninghaus W. Why we like to watch sad films. The pleasure of being moved in aesthetic experiences. Psychol Aesthetics Creat Arts. 2014;8(2):130–43. doi: 10.1037/a0035690

[93] Menninghaus W, Wagner V, Hanich J, Wassiliwizky E, Jacobsen T, Koelsch S. The Distancing-Embracing model of the enjoyment of negative emotions in art reception. Behav Brain Sci. 2017;40. doi: 10.1017/s0140525x17000309 28215214

[94] Engen HG, Singer T. Compassion-based emotion regulation up-regulates experienced positive affect and associated neural networks. Soc Cogn Affect Neurosci. 2015;10(9):1291–301. doi: 10.1093/scan/nsv008 25698699 PMC4560943

[95] Fredrickson BL. The role of positive emotions in positive psychology. The broaden-and-build theory of positive emotions. Am Psychol. 2001;56(3):218–26. doi: 10.1037//0003-066x.56.3.218 11315248 PMC3122271

[96] Martin L, Oepen R, Bauer K, Nottensteiner A, Mergheim K, Gruber H, et al. Creative Arts Interventions for Stress Management and Prevention-A Systematic Review. Behav Sci (Basel). 2018;8(2):28. doi: 10.3390/bs8020028 29470435 PMC5836011

[97] Abbing A, Ponstein A, van Hooren S. The effectiveness of art therapy for anxiety in adults: A systematic review of randomised and non-randomised controlled trials. Front Psychol. 2018;13:59–65. 10.1371/journal.pone.0208716 PMC6296656 30557381

[98] Hayes SC, Luoma JB, Bond FW, Masuda A, Lillis J. Acceptance and commitment therapy: model, processes and outcomes. Behav Res Ther. 2006;44(1):1–25. doi: 10.1016/j.brat.2005.06.006 16300724

[99] Bond FW, Hayes SC, Baer RA, Carpenter KM, Guenole N, Orcutt HK, et al. Preliminary Psychometric Properties of the Acceptance and Action Questionnaire–II: A Revised Measure of Psychological Inflexibility and Experiential Avoidance. Behav Ther. 2011;42(4):676–88. doi: 10.1016/j.beth.2011.03.007 22035996

[100] Batson CD, Early S, Salvarani G. Perspective Taking: Imagining How Another Feels Versus Imaging How You Would Feel. Personal Soc Psychol Bull. 1997;23:751–8.

[101] Thomson LJ, Lockyer B, Camic PM, Chatterjee HJ. Effects of a museum-based social prescription intervention on quantitative measures of psychological wellbeing in older adults. Perspect Public Health. 2018;138(1):28–38. doi: 10.1177/1757913917737563 29130869

[102] Anđelković Ž, Kovačić S, Bratić M, Vujičić MD, Stankov U, Pavluković V, et al. Museum Tour Guide Performance: A Visitor Perspective. Sustainability. 2022;14(16):10269. doi: 10.3390/su141610269

[103] Szubielska M, Imbir K. The aesthetic experience of critical art: The effects of the context of an art gallery and the way of providing curatorial information. PLoS One. 2021;16(5):e0250924. doi: 10.1371/journal.pone.0250924 34048445 PMC8162635

