# Supplementary material for: Art-induced psychological well-being: Individual traits shape the beneficial effects of aesthetic experiences
Source: PLoS One. 2025 Nov 14;20(11):e0332321. doi: 10.1371/journal.pone.0332321 (PMC12617907; doi:10.1371/journal.pone.0332321)
Supplement: S1 File — S1 Table. Ad-hoc questionnaire for evaluation of the visit. The table shows the questionnaire items, in their original Italian version (first column) and their English translation (second column), in the order they were presented to the participants. Participants had to evaluate each statement on a Likert scale from 1 to 7, where 1 corresponded to “Strongly disagree,” 2 to “Disagree,” 3 to “Somewhat disagree,” 4 to “Neither agree nor disagree,” 5 to “Somewhat agree,” 6 to “Agree,” and 7 to “Strongly agree”. S1 Fig. Post-visit evaluations. Each bar shows the mean score for an item from the post-visit questionnaire (full item statements are listed in S1 Table in S1 File). Items are arranged in descending order based on their scores. (DOCX) [file pone.0332321.s001.docx]

**SUPPORTING INFORMATION**

**Table S1. Ad-hoc questionnaire for evaluation of the visit.** The table shows the questionnaire items, in their original Italian version (first column) and their English translation (second column), in the order they were presented to the participants. Participants had to evaluate each statement on a Likert scale from 1 to 7, where 1 corresponded to "Strongly disagree," 2 to "Disagree," 3 to "Somewhat disagree," 4 to "Neither agree nor disagree," 5 to "Somewhat agree," 6 to "Agree," and 7 to "Strongly agree."


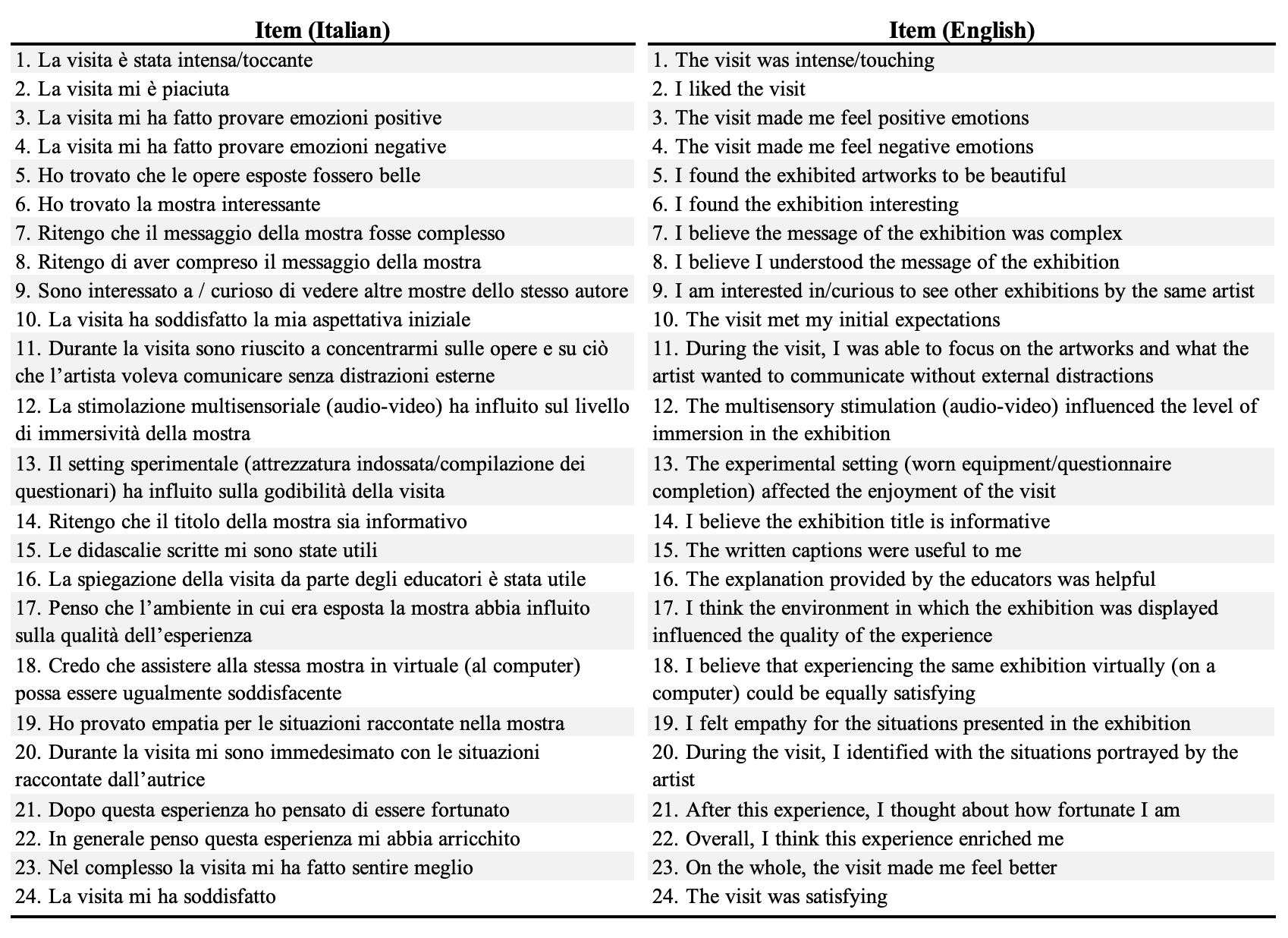


*
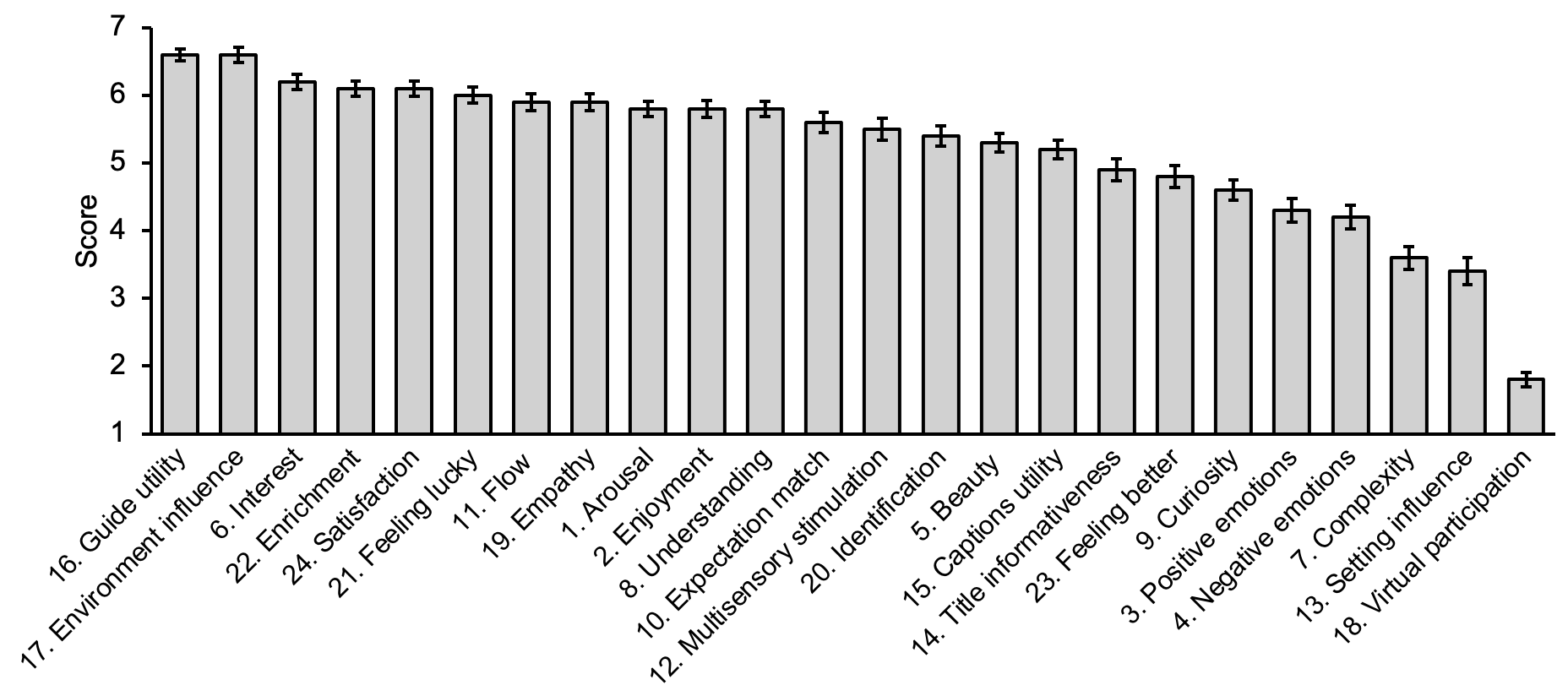
*

**Fig S1.** **Post-visit evaluations.** Each bar shows the mean score for an item from the post-visit questionnaire (full item statements are listed in Supplementary Table 1). Items are arranged in descending order based on their scores.
